# Onion (*Allium cepa* L.) Skin Waste Valorization: Unveiling the Phenolic Profile and Biological Potential for the Creation of Bioactive Agents through Subcritical Water Extraction

**DOI:** 10.3390/antiox13020205

**Published:** 2024-02-05

**Authors:** Esther Trigueros, Óscar Benito-Román, Andreia P. Oliveira, Romeu A. Videira, Paula B. Andrade, María Teresa Sanz, Sagrario Beltrán

**Affiliations:** 1 REQUIMTE/LAQV, Laboratory of Pharmacognosy, Chemical Department, Faculty of Pharmacy, University of Porto, R. Jorge Viterbo Ferreira, 228, 4050–313 Porto, Portugal; asoliveira@ff.up.pt (A.P.O.); rvideira@ff.up.pt (R.A.V.); pandrade@ff.up.pt (P.B.A.); 2 Department of Biotechnology and Food Science, Chemical Engineering Division, University of Burgos, Plza. Misael Bañuelos s/n, 09001 Burgos, Spain; obenito@ubu.es (Ó.B.-R.); tersanz@ubu.es (M.T.S.); beltran@ubu.es (S.B.)

**Keywords:** hydrothermal treatment, onion waste, polyphenols, quercetin, antioxidant activity, antidiabetic potential

## Abstract

Onion skin waste (OSW), the primary non-edible byproduct from onion processing, offers a renewable source of bioactive compounds. This study aims to valorize OSW through subcritical water extraction (SWE), aligning with a circular economy and biorefinery principles. SWE was carried out at 145 °C and 50 bar for 50 min in a discontinuous reactor, producing a phenolic-rich extract (32.3 ± 2.6 mg/g) dominated by protocatechuic acid (20.3 ± 2.5 mg/g), quercetin-4′-*O*-glucoside (7.5 ± 0.2 mg/g), and quercetin (3.2 ± 0.6 mg/g). Additionally, the extract contains sugars (207.1 ± 20.3 mg sucrose-Eq/g), proteins (22.8 ± 1.6 mg BSA-Eq/g), and free amino acids (20.4 ± 1.2 mg arginine-Eq/g). Its phenolic richness determines its scavenging activity against ^●^NO and O_2_^●−^ radicals and its α-glucosidase and aldose-reductase inhibition without affecting α-amylase. Notably, the extract demonstrates significant α-glucosidase inhibition (IC_50_ = 75.6 ± 43.5 µg/mL), surpassing acarbose (IC_50_ = 129.5 ± 1.0 µg/mL) in both pure enzyme and cell culture tests without showing cytotoxicity to AGS, HepG2, and Caco-2 human cell lines. The extract’s bioactivity and nutritional content make it suitable for developing antioxidant and antidiabetic nutraceutical/food components, highlighting SWE’s potential for OSW valorization without using organic solvents.

## 1. Introduction

Onions (*Allium cepa* L.) are one of the most widely consumed vegetables in the world, with an annual production of 93.23 million tons [1]. Onion processing generates significant non-edible waste; for instance, 0.6 million tons of onion waste is produced annually in Europe [2]. Unless appropriately managed, these residues could represent an environmental problem; however, they are also a source of bioactive compounds [1]. Onion skin, in particular, contains a higher concentration of flavonoids, notably quercetin [3], compared to other parts of the onion [4]. This may be due to the protective role of onion skin against soil microorganisms [5] and an enhanced flavonoid synthesis from protection against UV light exposure [2]. Phenolic acids like protocatechuic and *p*-coumaric acids, as well as flavonoids like kaempferol and isorhamnetin and various quercetin glucosides (quercetin-3-*O*-glucoside, quercetin-4′-*O*-glucoside, and quercetin-3,4′-*O*-diglucoside), are also found in onion skin [1]. These compounds are documented for their beneficial impacts on human health, exhibiting antioxidant, anticancer, antidiabetic, antiplatelet, anti-inflammatory, antibacterial, and neuroprotective properties [3]. 

Diabetes, one of the most prevalent diseases worldwide [6], involves the dysregulation of proteins, lipids, and the carbohydrate metabolism, resulting in elevated blood glucose levels [7]. Type 2 diabetes, constituting 90% of cases [8], is characterized by a reduced insulin action, leading to decreased glucose uptake by the liver, skeletal muscles, and fat cells [9]. Treatment aims to control blood glucose by stimulating insulin secretion and/or reducing insulin need [10], often involving the inhibition of carbohydrate digestion enzymes like α-amylase and α-glucosidase [11]. Common oral antidiabetics, such as acarbose, miglitol, and voglibose, are associated with numerous side effects, such as abdominal distention, flatulence, diarrhea, and liver disorders [12], which are related to strong α-amylase inhibition. Therefore, there is a search for natural compounds with potent α-glucosidase inhibition and a minimal impact on α-amylase. Onion skin, rich in phenolic compounds, holds potential for developing nutraceutical components to complement existing diabetes therapies [1].

To ensure safety for human consumption, the use of aqueous/ethanol solvents is preferred for extracting quercetin and other phenolic compounds [13]. Consequently, subcritical water extraction (SWE), a green technology based on using water within the temperature range of 100 to 374 °C and using a sufficient pressure to maintain a liquid state, has emerged as an excellent and safe solvent process for obtaining enriched phenolic extracts from onion skin that are suitable for incorporation into human nutrition. Under these conditions, water exhibits unique properties, including higher ionization and lower dielectric constants, facilitating the solubilization of different organic compounds [14]. 

SWE has been extensively documented for its ability to extract different bioactive compounds from plant-based raw materials [15,16,17]. Additionally, subcritical water has been employed to obtain polyphenols from various byproducts including purple sweet potato peels, cocoa pod husks, spent black tea and mango peel [18], chestnut peel [19], and pecan shells [20], among others. 

Although some studies have explored subcritical water extraction for extracting phenolic compounds from onion skin [21], there is a lack of research on the antidiabetic properties of these extracts. Hence, this research aims to valorize onion skin waste through subcritical water extraction, focusing on its potential for the development of nutraceuticals with antioxidant and antidiabetic activities. The evaluation includes analyzing the extract’s phenolic composition, antioxidant activity, and its ability to inhibit enzymes crucial in diabetes therapy. The results are then used to establish a correlation between the phenolic profile, antioxidant activity, and antidiabetic potential of the extract.

## 2. Materials and Methods

### 2.1. Chemicals

Aluminum chloride, ammonium acetate, methanol, NADH, nitrotetrazolium blue chloride (NBT), phenazine methosulfate (PMS), sodium nitroprusside dihydrate (SNP), N-(1-naphthyl)ethylenediamine dihydrochloride, potassium phosphate monobasic, sulfanilamide, D,L-glyceraldehyde, 2-mercaptoethanol, trypan blue solution (0.4%), starch, 3,5-dinitrosalicylic acid (DNS), 4-nitrophenyl-α-D-glucopyranoside (NPG), ninhydrin, potassium sodium tartrate tetrahydrate, potassium hydroxide, phosphoric acid, sodium phosphate, and sulfuric acid (95–97%) were supplied by Sigma-Aldrich (St. Louis, MO, USA), as well as acarbose (95%), quercetin (95%), BSA (96%), sucrose (≥99.0%), and arginine (≥98%), used as standard and positive controls. Ammonium sulfate was purchased from Merck (Darmstadt, Germany); FBS, Dulbecco’s Modified Eagle Medium DMEM (1X) + GlutaMAX, AB “Pen Strep” Glico, HBSS, and trypsin were purchased from GibcoTM, Thermo Fisher (Waltham, MA, USA); NADPH was purchased from Prozomix (Northumberland, UK); 3-(4,5-dimethylthiazolyl-2)-2,5-diphenyltetrazolium bromide (MTT) was purchased from Alfa Aesar Chemicals (Haverhill, MA, USA); dimethyl sulfoxide and acetonitrile were purchased from Carlo Erba Reagents (Val de Reuil, France); acetic acid was purchased from Atom Scientific; and 2-propanol, ethanol, sodium chloride, and sodium hydroxide were purchased from Honeywell (Seelze, Germany). The enzymes used for the in vitro enzymatic assays were aldose-reductase (*Homo sapiens*, human) from Prozomix (Northumberland, UK) and α-amylase (porcine pancreas) and α-glucosidase (*Saccharomyces cerevisiae*) from Sigma-Aldrich (St. Louis, MO, USA). Human gastric adenocarcinoma: AGS (ATCC CRL-1739), human colorectal adenocarcinoma: Caco-2 (ATCC HTB-27), and human liver hepatocellular carcinoma: HepG2 (ATCC HB-8065) cells were obtained from ATCC (Barcelona, Spain).

### 2.2. Plant Material

The onion skin waste (OSW) from the *Horcal* cultivar, harvested in 2021 in Burgos, Spain, was kindly provided by “Embutidos Cardeña” (Burgos, Spain). The outer skins were separated, air-dried (22 °C), and milled using a SM100 Retsch mill with a 1 mm sieve, resulting in an onion skin powder size lower than 1 mm. 

### 2.3. Subcritical Water Extraction

**Figure 1:**
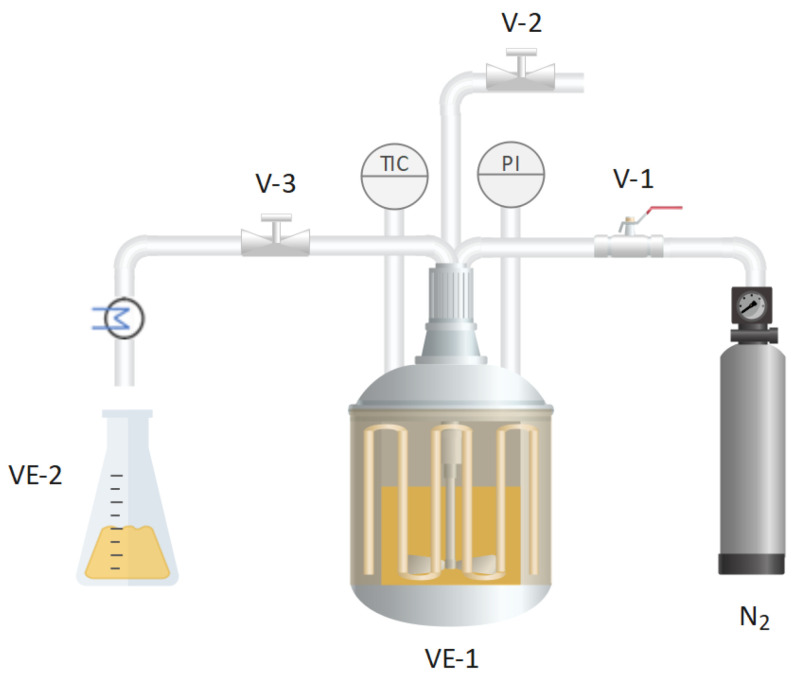
Scheme of the subcritical water equipment: reactor (VE-1), sample collector (VE-2), pressurization valve (V-1), pressure relief valve (V-2), and needle valve (V-3).

Subcritical water extraction was conducted in a 500 mL batch reactor (maximum pressure 7 MPa, maximum temperature 250 °C) under the conditions of 145 °C and 50 bar for 50 min, aligning with the optimal previous results obtained in our group [21]. In each experiment, 350 mL of water and 15 g of OSW were used, with the suspension in the reactor being stirred by a magnetic stirring bar. The system was heated using a ceramic electric band heater (2000 W) and pressurized with N_2_. The equipment, detailed in previous work [22], is shown in Figure 1. After cooling to 90 °C, the reactor was opened and the suspension was filtered. The spent onion skin was discarded, and the liquid was frozen at −80 °C, followed by freeze-drying (Labconco Freeze Dry System) at 0.15 mbar for at least 48 h to obtain a dry powder. The resulting extract was stored at −20 °C and protected from light to prevent sample oxidation. To conduct the assays described below, the freeze-dried extract was redissolved in the appropriate solvent required for each determination, henceforth referred to as the OSW-SWE extract. 

### 2.4. Chemical Characterization

#### 2.4.1. HPLC-DAD Analysis

The OSW-SWE extract was characterized by High-Performance Liquid Chromatography using a Diode Array Detector (HPLC-DAD, Agilent 1100, Santa Clara, CA, USA) and a Kinetex^®^ Biphenyl column (250 × 4.6 mm, particle size 5 µm, pore size 100 Å) from Phenomenex (Torrance, CA, USA). The mobile phase consisted of (A) ammonium acetate 5 mM with acetic acid in water (1%, *v*/*v*) and (B) ammonium acetate 5 mM with acetic acid in acetonitrile (1%, *v*/*v*). Elution began with 2% of (B) from 0 to 7 min, followed by a gradient to obtain 8% of (B) at 20 min, 10% of (B) at 35 min, 18% of (B) at 55 min, 38% of (B) at 65 min, 65% of (B) at 75 min, and 80% of (B) at 80 min. The post time was 10 min. The flow rate was set to 0.8 mL/min, and the column temperature was maintained at 25 °C. The sample was filtered (0.45 μm) before injection. Chromatograms were recorded at 280 nm, 330 nm, and 370 nm, and OpenLab CDS software (version 2.8) was used for data collection and analysis. Identification was achieved by comparing retention times and UV spectra with reference standards, and quantification was based on calibration curves obtained from these standards. Standard solutions were prepared and diluted with HPLC-grade methanol. At least five different concentrations were injected and analyzed in triplicate. The sample was diluted, when necessary, to fit to the calibration curves of standards (Table 1) and analyzed in triplicate.

#### 2.4.2. Total Flavonoid Content (TFC)

For TFC determination, the freeze-dried extract was redissolved in water to prepare a stock extract solution (5 mg/mL), and then diluted five times. Briefly, 0.5 mL of this diluted extract was mixed with 1.5 mL of ethanol, 0.1 mL of CH_3_COOK (0.1 M), 0.1 mL of AlCl_3_ (10%, *w*/*v*), and 2.8 mL of distilled water. After incubation for 30 min and filtration (0.45 μm), the absorbance at 415 nm was recorded. The reagents were prepared in distilled water, and the sample was analyzed in triplicate. A quercetin standard was utilized to calculate the TFC, expressed as milligrams of quercetin equivalent per gram of dry extract (mg QEq/g). A stock solution (1 mg/mL) was utilized to prepare five quercetin concentrations (0–500 µg/mL) for constructing the calibration curve, analyzing each point in triplicate. To adjust for the intrinsic color of the sample, a blank was prepared substituting 0.1 mL of AlCl_3_ by water, and the corresponding absorbance value was subtracted from the sample absorbance reading [23]. 

#### 2.4.3. Total Soluble Sugars

Total soluble sugars were quantified using the phenol–sulfuric method with some modifications [24]. In summary, the sample (100 µL) was combined with a phenol solution (100 µL) in sulfuric acid (6.5%, *v*/*v*) (500 µL), and absorbance was measured at 490 nm. The calibration curve was generated with sucrose as a standard. A water-based stock solution of sucrose (625 µM) was prepared, and different volumes were added to obtain standards containing 0–62.5 nmol. Six points with varying nmol contents were analyzed; each point was analyzed three times. The sample, initially dissolved in distilled water to achieve a concentration of 68.7 mg/mL and henceforth referred to as the ‘sample stock solution’, required a hundredfold dilution to fit the calibration curve. Three replicates were analyzed, and the results are expressed as mg of sucrose equivalent/g extract. 

#### 2.4.4. Total Protein Content

The Bradford method [25] was employed to determine the total protein content in the OSW-SWE extract. In short, 40 µL of the sample and 200 µL of Bradford reagent were incubated in a 96-well plate for 5 min in the dark. The absorbance was then measured at 595 nm. A reference standard of BSA in distilled water (1 mg/mL) was prepared, and various volumes were added to create a calibration curve with six concentration points (0–50 µg/mL). Each point was analyzed in triplicate. The sample stock solution was diluted fifty times to align with the calibration curve and analyzed in triplicate. The results are expressed as mg of BSA equivalent/g extract. 

#### 2.4.5. Total Free Amino Acid Content

Free amino acids were quantified using the ninhydrin reaction method from Sigma Aldrich (Saint Louis, MO, USA) with some modifications. A ninhydrin solution (0.1 M) was prepared in acetone and sodium acetate buffer (100 mM, pH 5.0) under a nitrogen atmosphere. Briefly, the sample (500 µL) was combined with a ninhydrin solution (500 µL), incubated in a boiling water bath for 10 min, followed by cooling and the addition of 1 mL of ethanol. Finally, the absorbance was measured at 570 nm [14]. A calibration curve comprising six points ranging from 0 to 1 mmol of arginine was constructed using a 20 mM stock solution in distilled water. To bring the protein concentration of the sample within the calibration curve range, the sample stock solution was diluted tenfold and then analyzed in triplicate. The results are expressed as mg of arginine equivalent/g extract.

### 2.5. Radical Scavenging Activity

To assess the antioxidant activity of the OSW-SWE extract, the freeze-dried extract was re-dissolved in water, in a volume allowing to obtain a solution of known concentration. If needed, the sample solution was diluted to cover the broadest range of inhibitory activities for constructing inhibition curves and estimating IC_50_ values. A minimum of five concentrations were tested, and at least three independent experiments were conducted in triplicate.

#### 2.5.1. Nitric Oxide (^●^NO) Scavenging Activity

Determination of ^●^NO scavenging activity was performed by adding equal volumes (75 µL) of sample and SNP (20 mM) and exposing it to light for 60 min. Subsequently, the same volume of Griess reagent, composed of a mixture of sulfanilamide and *N*-(1-naphthyl) ethylenediamine dihydrochloride in phosphoric acid (2%, *v*/*v*), was added and kept in the dark for 10 min. Finally, the absorbance was measured at 560 nm [26]. All the reagents were prepared in phosphate buffer (0.1 M, pH 7.4). A control was prepared with buffer instead of the sample, and the scavenging activity was calculated as the reduction in absorbance caused by the sample compared to control. A solution of quercetin in DMSO (1 mg/mL) was used as a positive control.

#### 2.5.2. Superoxide (O_2_^●−^) Scavenging Activity

To determine the O_2_^●−^ scavenging activity, equal volumes (50 µL) of the sample, NADH (166 µM), PMS (2.7 µM), and triple the amount (150 µL) of NBT (43 µM) were mixed, and the absorbance was recorded at 560 nm [26]. A control was prepared replacing the sample with buffer, and the results were expressed as scavenging activity relative to the control. Phosphate buffer (19 mM, pH 7.4) was used to prepare reagent solutions and the control and to dilute the sample. A solution of quercetin in DMSO (0.3 mg/mL) served as a positive control.

### 2.6. In Vitro Enzymatic Assays

In order to assess the antidiabetic effects of the OSW-SWE extract, the same procedure as in Section 2.5 was employed to redissolve the extract. Also, a minimum of five concentrations and three independent experiments were conducted in triplicate.

### 2.6.1. α-Amylase Inhibition Assay

To estimate the α-amylase activity, equal volumes (100 µL) of sample, starch solution in water (1%, *w*/*v*), and α-amylase (≥5 U/mg) were incubated for 10 min at 25 °C. Then, 200 µL of DNS was added, and the mixture was incubated at 100 °C for 5 min. After cooling, the absorbance was measured at 540 nm. A control, prepared with phosphate buffer (pH 6.9) instead of the sample, was used for enzyme inhibition quantification [26]. An aqueous solution of acarbose (1 mg/mL) was used as the positive control.

### 2.6.2. α-Glucosidase Inhibition Assay

α-Glucosidase activity was evaluated according to a previous method with some modifications [26]. The assay was performed at 37 °C in potassium phosphate buffer (10 mM, pH = 7.0) with 0.0233 U/mL of enzyme and NPG 833.33 µM. The reaction, initiated by adding 100 µL of NPG to a mixture of sample (50 µL), buffer (130 µL), and enzyme (20 µL), was monitored for 10 min by measuring the increase in absorbance at 405 nm, indicating the release of yellow 4-nitrophenol. Acarbose (0.5 mg/mL) in water was used as a positive control.

### 2.6.3. Aldose Reductase Inhibition Assay

The aldose reductase activity was assessed following a previous method [26]. Briefly, equal volumes (40 µL) of sample, enzyme (1 mg/mL), and D,L-glyceraldehyde (10 mM) were added in a 96-well plate. A control was prepared with phosphate buffer instead of the sample. The plate was preincubated at 37 °C for 2 min, and the reaction was initiated by adding 80 µL of NADPH (0.5 mM), reading the absorbance at 340 nm immediately. Subsequently, it was incubated for 20 additional minutes and the absorbance was measured. All the reagents were prepared in phosphate buffer (0.1 M, pH 6.2) with ammonium sulfate (0.2 mM) and 2-mercaptoehanol (5 mM). Quercetin solution (0.5 mg/mL) in water served as the positive control.

## 2.7. Cellular Assays

### 2.7.1. Cell Culture

AGS, Caco-2, and HepG2 cells were thawed and cultured in DMEM (1X) + GlutaMAX medium supplemented with FBS (10%, *v*/*v*) and penicillin/streptomycin (1%, *v*/*v*). The cells were maintained at 37 °C in a humidified atmosphere with 5% CO_2_ and split twice before use. After reaching 80–90% confluence, cells were washed with HBSS, trypsinized, and subcultured on 96-well plates as detailed below.

### 2.7.2. Cell Viability Assay

The effects of the OSW-SWE extract on cell viability were assessed through an MTT reduction assay [26]. Cells were seeded at densities of 15,000, 20,000, and 10,000 cells/well for AGS, Caco-2, and HepG2, respectively, and incubated at 37 °C for 24 h. Then, cells were incubated with the extract at various concentrations for 24 h. For this purpose, the freeze-dried extract was re-dissolved in DMSO to reach a concentration of 68.7 mg/mL and diluted 200 times in the medium to maintain a non-toxic DMSO concentration (≤0.5%). After removing the medium, MTT was added, and the absorbance was measured at 560 nm. A control with an equivalent proportion of DMSO in the medium was used to estimate cell survival. At least three independent experiments were performed in triplicate.

### 2.7.3. Inhibition of α-Glucosidase in Caco-2 Cells

The inhibitory effect of the OSW-SWE extract on the human α-glucosidase system (dimeric enzyme sucrase-isomaltase system) was assessed following the methodology described by Ferreres et al. [26] with some modifications. Caco-2 cells were cultured in T75 flasks, washed with HBSS, and collected with cold PBS containing a protease inhibitor cocktail. Cells were disrupted by using a glass/Teflon Potter Elvehjem and the homogenate was centrifugated at 12,500 g for 20 min. The pellet, consisting of the cell nucleus and other dense organelles, was discarded, whereas the supernatant, containing the plasma membrane vesicles with the dimeric enzyme sucrase–isomaltase system, was stored at −4 °C. The protein content of the supernatants was determined via the Bradford method with BSA as a standard, as previously described in Section 2.4.4. The sample dissolved in DMSO was diluted in the medium to reach a concentration of 500 µg/mL. Human α-glucosidase activity was measured using plasma-membrane-rich Caco-2 cell supernatants (protein concentration: 0.24 to 0.39 mg/mL) and 1000 µM NPG with increasing extract concentrations (0–500 µg/mL). Assays were conducted in potassium phosphate buffer (10 mM, pH = 7.0) at 37 °C for 4 h, monitoring the release of yellow 4-nitrophenol at 405 nm. The enzyme activity, normalized by protein content, was used to calculate enzyme inhibition as function of the extract concentrations. Three independent assays were performed for each concentration, using plasma-membrane-rich Caco-2 cell supernatants from distinct cell passages. Acarbose (129.5 µg/mL) was used as a positive control. Finally, the inhibitory effect of the OSW-SWE extract on the human α-glucosidase system in adherent Caco-2 cells was directly assessed. The extract was tested at the concentration required to reduce 50% the enzyme activity in plasma-membrane-rich Caco-2 cell supernatants (i.e., IC_50_ in the previous assay). Caco-2 cells, cultured until full confluence, were first washed and then incubated at 37 °C with HBSS containing NPG 821.31 µM in the absence (control) and presence of the extract. After two hours, the medium was collected to measure the yellow 4-nitrophenol released at 405 nm. Cells were collected with Triton X-100 buffer solution (2 g/100 mL) and used to assess cell protein contents. Blank assays were performed to account for the intrinsic color of cells and the extract. The α-glucosidase system activity, normalized by cell protein, was expressed as a percentage of the control. Assays were conducted in triplicate using different cell passages.

### 2.8. Statistical Analysis

GraphPad Prism 8.4.2 software was used for statistical analysis. All determinations were performed at least in triplicate and the results are expressed as means ± standard error of the mean. A Student’s *t*-test (unpaired-two tailed) was used to confirm significant differences between the extract and the control (*p*-value < 0.05), and between the IC_50_ values of the OSW-SWE extract and the positive control (*p*-value < 0.01).

## 3. Results and Discussion

### 3.1. Chemical Characterization

**Figure 2:**
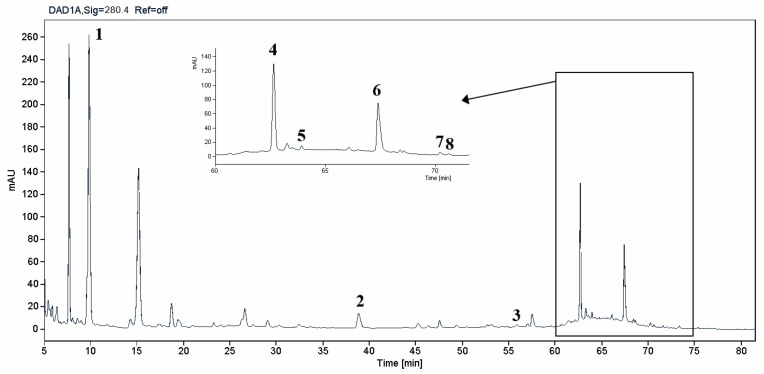
HPLC-DAD chromatogram of the extract obtained from *Horcal* onion skin waste through subcritical water extraction at 145 °C and 50 bar for 50 min. Determination at 280 nm. Compounds’ identities are shown in Table 2.

The HPLC-DAD analysis identified and quantified eight phenolic compounds in the OSW-SWE extract, comprising phenolic acids and flavonoids (Table 2 and Figure 2), including major constituents such as protocatechuic acid, quercetin-4′-*O*-glucoside, and quercetin, corresponding to ca. 62.5%, 23.4%, and 10% of the total phenolics identified, respectively. Conversely, *p*-coumaric acid, quercetin-3-*O*-glucoside, myricetin, kaempferol, and isorhamnetin exhibited a significantly lower content, collectively contributing to 1.3 mg/g. In agreement with HPLC data, the TFC of the OSW-SWE extract, assessed by a colorimetric assay, is 26 ± 3 mg QE/g. These data agree with the results reported in the literature. In one study, onion peel extracts obtained through SWE (165 °C) exhibited a TFC and a total quercetin content of 27.10 and 12.26 mg QE/g, respectively [27]. The TFC value closely resembled that obtained in this study, while the quercetin content was higher. This observation can be attributed to differences in the onion skin cultivar, harvest location, and/or the conditions employed during SWE. The cited study utilized onion skin fractions ranging from 1 to 10 mm, whereas our study used a fraction smaller than 1 mm. Additionally, variations in extraction conditions were evident. The referenced study employed a shorter treatment duration (15 min) and a higher temperature, which may be associated with the increased preservation of quercetin and a heightened degradation of other phenolic compounds, resulting in a similar TFC. As previously reported by our group [21], the quercetin content decreases when the SWE (145 °C) time exceeds 10–20 min. Moreover, it was observed that the quercetin content in the extract was higher at 160 °C compared to 145 °C when the treatment time ranged from 10 to 20 min. Other studies also confirmed the presence of quercetin, quercetin-4′-*O*-glucoside, and kaempferol in subcritical water extracts obtained from onion skin [28]. Apart from its phenolic composition, the OSW-SWE extract also includes other nutritional compounds such as sugars (207 ± 20 mg sucrose-Eq/g) and proteins (22.8 ± 1.6 mg BSA-Eq/g), along with free amino acids (20.4 ± 1.2 mg arginine-Eq/g), demonstrating water’s ability under subcritical conditions to hydrolyze the protein fraction.

### 3.2. Effects on Protection against Radicals

Multiple studies have associated insulin resistance with oxidative stress and complications associated with diabetes [12,29]. Insulin resistance, affecting the liver, skeletal muscles, and fat cells, leads to chronic hyperglycemia. Under this condition, the non-insulin-dependent glucose uptake tissues stimulate the polyol pathway, increasing aldose-reductase activity with a consequent NADPH depletion. Consequently, cells lose the capacity to promote the regeneration of reduced glutathione (GSH), increasing the GSSG/GSH ratio for values that increase cell susceptibility to oxidative damage. Simultaneously, sorbitol accumulation pushes the polyol pathway, generating fructose and potent glycating agents. These agents react with protein, lipids, and nucleic acids to form advanced glycation end products [30], which negatively affect cell function, compromising antioxidant defenses and enhancing oxidative stress and tissue damage. Thus, extracts from edible sources with antioxidant activity and competence to inhibit carbohydrate hydrolysis enzymes (e.g., α-glucosidases) and aldose reductase are promising tools to combat diabetes complications by reducing glucose absorption and mitigating oxidative damage and inflammation induced by hyperglycemia.

**Figure 3:**
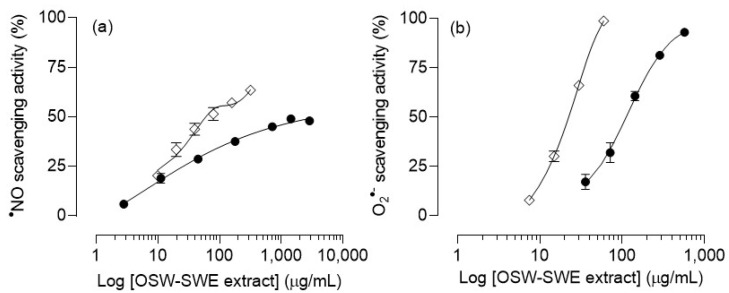
(**a**) Nitric oxide (^●^NO) and (**b**) superoxide anion (O_2_^●−^) radical scavenging activity of onion skin waste extract (●) obtained via subcritical water extraction (OSW-SWE) at 145 °C and 50 bar for 50 min, in comparison with quercetin (◇), used as a positive control and tested under the same experimental conditions as the sample. Data are expressed as means ± standard error of the mean (*n* = 3).

The OSW-SWE extract’s ability to scavenge ^●^NO and O_2_^●−^ radicals is shown in Figure 3. For ^●^NO, the extract exhibits a scavenging activity below 50% within the tested concentrations. Although an initial concentration-dependent trend is observed, a plateau is reached at higher concentrations, and the IC_50_ value cannot be determined (Figure 3a). Regarding the O_2_^●−^ scavenging activity, the extract demonstrates greater efficacy than for ^●^NO (Figure 3b). It shows a notable capacity to neutralize O_2_^●-^ (IC_50_ = 112 ± 7 µg/mL), but it is less active than quercetin (IC_50_ = 22.4 ± 1.1 µg/mL) (Table 3). This may attributed to the presence of other compounds, mainly protocatechuic acid, which has demonstrated a lower capacity to scavenge superoxide anion radicals compared to quercetin in its pure form (IC_50_ = 310.3 µg/mL) [31]. Thus, the OSW-SWE extract has been proven as an effective scavenging agent toward oxidant species like O_2_^●−^ and ^●^NO, despite exhibiting a lower activity than quercetin, likely due to the presence of other less active compounds in its composition. Quercetin is widely recognized for its antioxidant potential, effectively scavenging reactive oxygen species like O_2_^●−^, ^●^NO, and ONOO^-^ [29]. This capacity is related to the presence of -OH groups in its structure, facilitating the neutralization of free radicals.

While most of the available studies have assessed the antioxidant potential of onion skin extracts through DPPH, FRAP, ABTS, and ORAC assays [32], only a limited number have investigated their scavenging activity against oxidants causing damage to macromolecules in biological systems and in foodstuffs, such as O_2_^●−^ and ^●^NO. As an example, a study evaluated the antioxidant potential of onion skin extracts obtained using various solvents (*n*-butanol, ethyl acetate, methanol, ethanol, and distilled water). All extracts demonstrated DPPH scavenging activities above 60% at 100 µg/mL; however, their ability to scavenge ^●^NO was below 40% at the highest tested concentration (1600 µg/mL) [33]. Consequently, our OSW-SWE extract proves to be more effective against ^●^NO than those previously reported, with the concentration required to scavenge 40% of ^●^NO being lower than 300 µg/mL (Figure 3a).

### 3.3. Effects on Enzymatic Digestion of Carbohydrates

#### 3.3.1. α-Amylase and α-Glucosidase Inhibition

Various hydrolytic enzymes, including α-glucosidase and α-amylase, play roles in carbohydrate digestion. α-Amylase catalyzes the initial stage of starch hydrolysis by breaking the α-(1 → 4) glycosidic bonds, producing maltose, maltotriose, maltotetraose, maltodextrins, and glucose. On other hand, α-glucosidase is responsible for the final phase of starch digestion and the hydrolysis of disaccharides. Inhibiting these enzymes can slow carbohydrate digestion, preventing elevated blood glucose levels and postprandial hyperglycemia [11,34].

**Figure 4:**
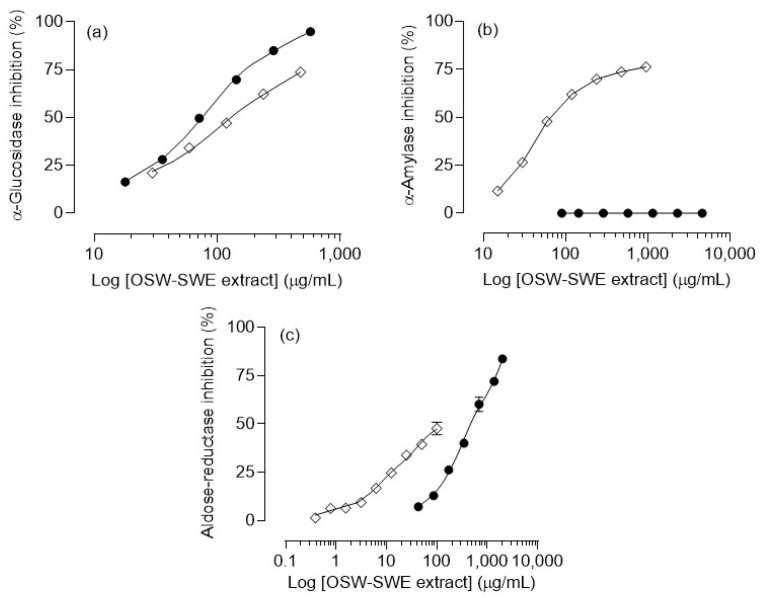
Inhibitory effects of the extract obtained from *Horcal* onion skin waste through subcritical water extraction (OSW-SWE) at 145 °C and 50 bar for 50 min (●) in comparison with the positive control (◇), tested under the same experimental conditions as the sample, on different enzymes involved in carbohydrate digestion: (**a**) α-glucosidase, (**b**) α-amylase, and (**c**) aldose-reductase. Acarbose was used as a positive control for α-glucosidase and α-amylase assays, and quercetin was used for the aldose-reductase assay. Data are expressed as means ± standard error of the mean (*n* = 3).

The OSW-SWE extract demonstrates a significant and concentration-dependent inhibitory effect on α-glucosidase activity, as depicted in Figure 4a. Notably, the extract exhibits potent inhibition, with an IC_50_ value of 76 ± 4 µg/mL (Table 3), significantly (*p* < 0.01) lower than that determined for the pharmacological inhibitor acarbose (129.5 ± 1.0 µg/mL) under identical experimental conditions. Concerning α-amylase activity, the OSW extract obtained via SWE does not disrupt the normal functioning of the enzyme (Figure 4b). This finding is significant, considering that various oral antidiabetic drugs, such as acarbose, miglitol, and voglibose, have been associated with adverse effects due to their intense α-amylase inhibition [12,34]. In this context, the tested extract emerges as a potential alternative for diet-based diabetes prevention/management approaches, offering robust α-glucosidase inhibition without interfering with the normal α-amylase activity, thereby avoiding the associated adverse effects. These findings may be related to the extract’s chemical composition. Protocatechuic acid, identified as the major compound, is known as an α-glucosidase inhibitor with IC_50_ values lower than acarbose and other phenols like *p*-coumaric acid [35]. Additionally, quercetin, also present in the extract, has been extensively studied for its antidiabetic effects, demonstrating the ability to inhibit α-glucosidase [29,36]. In terms of their impact on α-amylase activity, three phenolic characteristics have been identified: (1) the quantity of hydroxyl groups, (2) the presence of a conjugated double bound (C2-C3) in ring C along with a carbonyl group (C4) in ring A, and (3) the degree of flavonoid glycosylation. The first two lead to a reduction in the enzyme activity due to the higher number of hydroxyl groups and the higher ability to form hydrogen bonds with amino acids, such as aspartic and glutamic acids, at the enzyme’s active site. Additionally, the unsaturated rings can form a stable electron-rich system with the indole ring of the amino acid tryptophan at the active site of the enzyme. Conversely, flavonoid glycosylation diminishes the inhibitory effect on α-amylase, as bulky glucoside structures limit the hydroxyl group’s access to the enzyme’s active site [37]. Protocatechuic acid, the major compound determined in the OSW-SWE extract, may exhibit a reduced binding ability to the active center of the enzyme as it lacks the described ring system and presents only three -OH groups. In this context, it was reported that the inhibitory impact of pure protocatechuic acid is less pronounced than that of acarbose. Additionally, quercetin 4′-*O*-glucoside demonstrates a reduced binding capacity to the enzyme compared to quercetin due to the steric impediment posed by its bulky structure after undergoing glycosylation [35]. These results provide insights into the reason why the OSW-SWE extract does not affect α-amylase activity.

As far as we know, no studies have explored the potential of onion skin extracts obtained through subcritical water extraction to inhibit enzymes related to diabetes. Extracts obtained from other vegetal sources have shown α-glucosidase inhibition, but also a reduced α-amylase activity. For example, *Castanea mollissima* extracts obtained via SWE (from 120 to 200 °C) had IC_50_ values between 10 and 25 µg/mL for α-glucosidase, lower than those obtained for the OSW-SWE extract. Additionally, these extracts exhibited α-amylase inhibition, with IC_50_ values ranging from 15 to 50 µg/mL [38]. A pumpkin polysaccharide obtained via SWE showed antidiabetic potential but with lower inhibition rates (below 15%) [39]. Proanthocyanidins from Chinese quince fruits extracted through subcritical ethanol:water extraction, demonstrated high α-amylase inhibition rates, reaching up to 86% at 0.5 mg/mL [40]. This confirms the extract’s significant capacity to strongly inhibit α-glucosidase without affecting the α-amylase activity. The requirement for higher extract concentrations to reduce α-amylase activity demonstrates a valuable advantage in preventing adverse side effects associated with potent inhibition, while effectively slowing carbohydrate digestion.

#### 3.3.2. Aldose-Reductase Inhibition

Figure 4c illustrates the OSW-SWE extract’s concentration-dependent inhibition of aldose-reductase, with levels exceeding 80% at the highest concentration tested. However, the extract’s IC_50_ value is significantly higher (*p* < 0.01) than the value of quercetin (Table 3), used as positive control, attributed to the extract’s lower quercetin content. The results found in this study for aldose-reductase inhibition indicate that the OSW-SWE extract significantly outperforms other extracts reported in the literature. For example, extracts from various fruits and vegetables showed higher IC_50_ values, and an ethanolic extract from red onion exhibited concentration-dependent inhibition, but the activity was too weak to determine the IC_50_ value [41]. Consequently, the extract obtained in this work has the potential to be considered as a natural source of compounds to control blood glucose levels and oxidative damage and to manage the clinical complications associated with diabetes.

### 3.4. Effects on Cell Viability

To improve the potential of the OSW-SWE extract to support the development of nutraceutical/food components for type 2 diabetes management, it was crucial to assess its toxicological impact on human cells. The evaluation focused on AGS, Caco-2, and HepG2 cells’ viability, commonly employed as cellular models for studying the effects of bioactive compounds on the gastrointestinal epithelium, including absorption, metabolism, and toxicity, despite their status as cancer cell lines [1].

**Figure 5:**
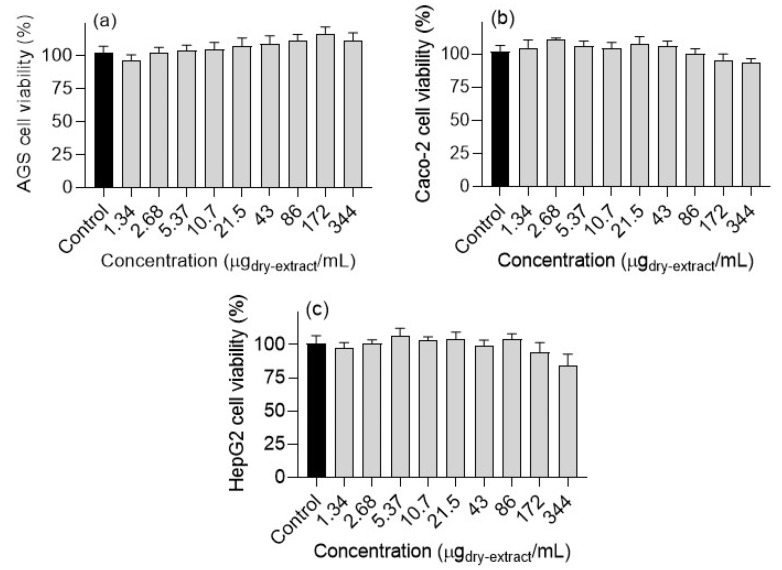
Effects of *Horcal* skin onion extracts obtained by means of subcritical water at 145 °C and 50 bar for 50 min on the viability of (**a**) AtGS, (**b**) Caco-2, and (**c**) HepG2 cells. Data are expressed as means ± standard error of the mean (*n* = 3).

Cell viability was evaluated via the MTT reduction assay. In the range of tested concentrations, the OSW-SWE extract does not cause significant changes to the cell viability of AGS, HepG2, and Caco-2 cell lines (Figure 5). Onion skin extracts are known for their ability to reduce cell survival in various human cancer cell lines [1]. For instance, the antiproliferative impact of edible pulp onion extracts was studied on HepG2 and Caco-2 cells, revealing a dose-dependent decrease in cell viability. However, the extracts were assayed at high concentrations, determining IC_50_ values exceeding 20 mg/mL for all the extracts and cell lines [42]. In contrast, our study tested concentrations up to a maximum of 344 µg/mL. Nevertheless, for a comprehensive understanding of the physiological significance of the orally administered extract, it is essential to consider the bioavailability of bioactive compounds reaching the target cells. For example, research indicates that quercetin glucosides are primarily absorbed in the small intestine, with approximately 48% of these compounds reaching and interacting with colon cells [42].

### 3.5. α-Glucosidase Inhibition in Caco-2 Cells

As previously revealed, the OSW-SWE extract effectively inhibits yeast α-glucosidase (Figure 4a), with an IC_50_ value lower than acarbose (Table 3). However, the efficacy of enzyme inhibition depends on the enzyme used in the assay. Enzymes from different biological sources (e.g., yeast, rats, humans) show differing sensitivity to inhibitors [43]. To evaluate the impact of the OSW-SWE extract on the human sucrase–isomaltase enzymatic system (α-glucosidases), assays were conducted using enzyme-enriched cell supernatants from homogenates of human Caco-2 cells. These cells are commonly used in pre-clinical assessment of human α-glucosidase inhibitors due to their resemblance to the human intestinal epithelium, including the α-glucosidase system’s expression in apical membranes [26].

**Figure 6:**
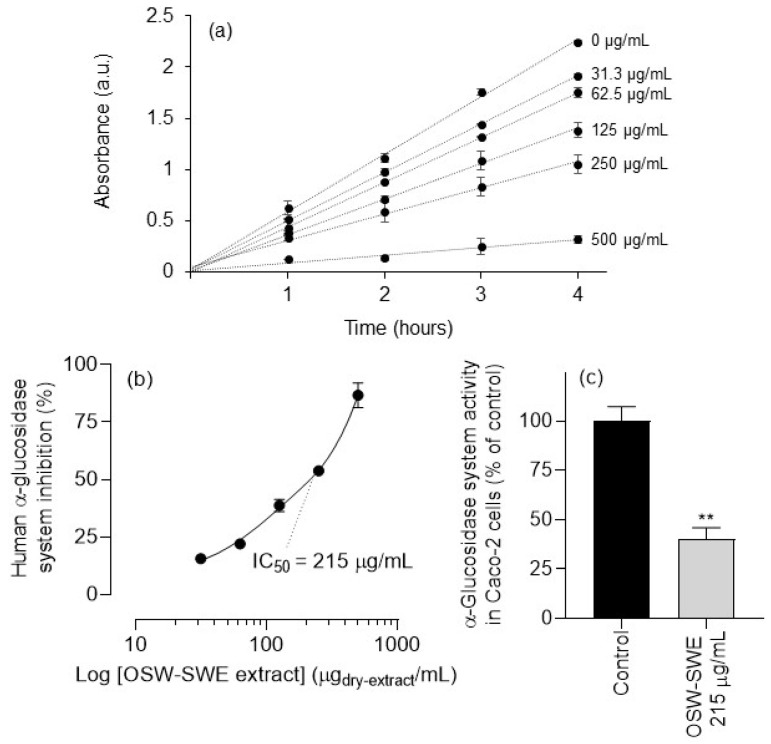
(**a**) Kinetics of NPG substrate conversion in the absence and presence of different concentrations of the OSW-SWE extract. (**b**) Inhibitory effect of the extract on supernatants from homogenates of the human α-glucosidase system from Caco2 cells as a function of log concentration of the extract in the presence of 5 mM of *p*-NPG. (**c**) Activity of the α-glucosidase system in adherent Caco-2 cells (100% of activity in control corresponds to 28 ± 6 nmol of *p*-nitrophenol released per hour and mg of cell protein). ** *p* < 0.01, significantly different from the control. Data are expressed as means ± standard error of the mean (*n* = 3).

Figure 6a depicts the substrate conversion kinetics of enzyme-enriched cell supernatants from homogenates of human Caco-2 cells in the absence and presence of varying concentrations of the OSW-SWE extract over 4 h. The OSW-SWE extract effectively inhibits the human α-glucosidase system, as shown in Figure 6b, with an estimated IC_50_ value of 215 ± 12 µg/mL, nearly three times the value observed with yeast enzyme (Figure 4a). Furthermore, compared to acarbose, the extract exhibits a stronger inhibitory activity on the human α-glucosidase system, as acarbose at 129.5 µg/mL (its IC_50_ value for yeast α-glucosidase) causes only a 12.3 ± 1.3% inhibition. Additionally, the OSW-SWE extract shows inhibition in the human α-glucosidases system within a concentration range that shows no signs of toxicity for Caco-2, AGS, and HepG2 cells (Figure 5).

The competence of the OSW-SWE extract to inhibit the human α-glucosidase system was further evaluated in adherent Caco-2 cells using a concentration corresponding to its IC_50_ value, estimated in the α-glucosidase system of plasma membrane-rich Caco-2 cell supernatants (Figure 6b). Figure 6c illustrates a significant (*p* < 0.01) inhibition of the α-glucosidase system in adherent Caco-2 cells. This result not only confirms the extract’s enzyme inhibition potency, as observed in plasma-membrane-rich supernatants of Caco-2 cell homogenates, but also suggests that the α-glucosidases inhibitors in the OSW-SWE extract remain in the extracellular medium for at least two hours, indicating limited metabolism and/or uptake by Caco-2 cells.

## 4. Conclusions

Subcritical water extraction technology proves to be a suitable approach for converting onion skin waste into a valuable renewable resource to develop new food/nutraceutical components targeting oxidative damage and diabetes. This method yields an extract with distinctive nutritional and phenolic compounds, showcasing a significant antioxidant capacity. Moreover, the extract demonstrates heightened inhibition of α-glucosidase compared to acarbose, without affecting α-amylase activity, potentially mitigating the side effects associated with common oral antidiabetics drugs. The non-cytotoxic nature of the extract, along with these characteristics, highlights subcritical water extraction as an effective technology for valorizing onion skin waste and producing bioactive and phenol-rich extracts.

## Figures and Tables

**Table 1:** Linear regression parameters, limit of detection (LOD), and quantification (LOQ) for standards of phenolic compounds quantified by HPLC-DAD analysis.

Phenolic Standard	Wavelength = 280 nm				
	Regression Equation	*R* ^2^	Concentration Range (µg)	LOD (µg)	LOQ (µg)
Protocatechuic acid	y = 1721.4x + 61.2	0.9984	0.072–8.582	0.022	0.067
*p*-Coumaric acid	y = 5839.2x +187.2	0.9990	0.034–13.0	0.010	0.030
Quercetin-3-*O*-glucoside	y = 1218.3x − 8.6	0.9998	0.020–2.61	0.006	0.018
Quercetin-4′-*O*-glucoside	y = 1310.3x + 14.9	0.9998	0.050–5.94	0.015	0.045
Myricetin	y = 1535.4x − 70.9	0.9994	0.268–2.679	0.030	0.090
Quercetin	y = 1578.5x + 165.1	0.9990	0.127–15.81	0.030	0.090
Kaempferol	y = 2036.4x − 3.4	0.9999	0.073–2.273	0.020	0.060
Isorhamnetin	y = 3877x + 106.0	0.9980	0.063–3.136	0.006	0.018
Phenolic Standard	**Wavelength = 330 nm**				
	Regression equation	*R* ^2^	Concentration range (µg)	LOD (µg)	LOQ (µg)
Protocatechuic acid	nd	nd	nd	nd	nd
*p*-Coumaric acid	y = 4923.4x + 362.1	0.9983	0.037–13	0.010	0.030
Quercetin-3-*O*-glucoside	y = 1992x − 16.6	0.9999	0.02–2.61	0.006	0.018
Quercetin-4′-*O*-glucoside	y = 1793.7x + 29.5	0.9998	0.0397–5.94	0.010	0.030
Myricetin	y = 2099.4x − 131.3	0.9994	0.268–2.679	0.030	0.090
Quercetin	y = 2012.4x + 322.9	0.9969	0.067–15.81	0.020	0.060
Kaempferol	y = 2792.7x − 6.3	0.9999	0.033–2.273	0.010	0.030
Isorhamnetin	y = 4892.2x + 233.9	0.9956	0.063–6.272	0.006	0.018
Phenolic Standard	**Wavelength = 370 nm**				
	Regression equation	*R* ^2^	Concentration range (µg)	LOD (µg)	LOQ (µg)
Protocatechuic acid	nd	nd	nd	nd	nd
*p*-Coumaric acid	nd	nd	nd	nd	nd
Quercetin-3-*O*-glucoside	y = 2199.6x − 7.4	0.9965	0.099–2.61	0.003	0.010
Quercetin-4′-*O*-glucoside	y = 2778.2x + 132.3	0.9994	0.030–5.94	0.006	0.018
Myricetin	y = 4440.6x − 112.9	0.9999	0.268–2.679	0.010	0.030
Quercetin	y = 3024.4x + 1677.8	0.9900	0.022–15.81	0.006	0.018
Kaempferol	y = 4897.9x + 24.1	0.9999	0.023–2.273	0.005	0.015
Isorhamnetin	y = 9324.1x + 462.2	0.9943	0.063–1.568	0.002	0.006

*R*^2^: correlation coefficient; LOD: limit of detection; LOQ: limit of quantification; nd: not detected.

**Table 2:** The chemical profile of phenolic compounds identified by HPLC-DAD from onion skin waste extract (OSW-SWE) obtained by subcritical water extraction at 145 °C and 50 bar for 50 min. Results are expressed as mg per gram of dry extract. * Data are expressed as means ± mean standard error (*n* = 3).

Phenolic Compounds			OSW-SWE			
			Rt (min)	(mg/g_dry-extract_) *		
1	Protocatechuic acid	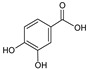	9.7	20.3	±	2.5
2	*p*-Coumaric acid	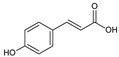	39.9	0.10	±	0.05
3	Quercetin-3-*O*-glucoside	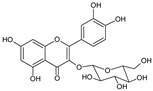	55.5	0.10	±	0.05
4	Quercetin-4′-*O*-glucoside	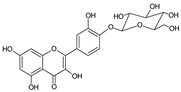	62.6	7.5	±	0.2
5	Myricetin	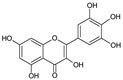	63.8	0.7	±	0.1
6	Quercetin	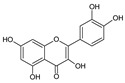	67.5	3.2	±	0.6
7	Kaempferol	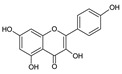	70.1	0.20	±	0.05
8	Isorhamnetin	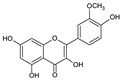	70.6	0.20	±	0.05
∑ phenolic compounds (mg/g_dry-extract_)				32.3	±	2.6
TFC (mgQEq/g_dry-extract_) (colorimetric assay)				26.0	±	3.0

**Table 3:** IC_50_ values determined for the extract obtained from onion skin waste in radical scavenging and pure enzyme activity determination, in comparison with those determined for positive controls. Data are expressed as means ± mean standard error (*n* = 3).

	IC_50_ (µg/mL) *^1^	
Assay	OSW—SWE	Positive Control
**Free radical scavenging**		
Nitric oxide	>2863 *^2^	60 ± 2
Superoxide	112 ± 7 ^a^	22.4 ± 1.1 ^b^
**Pure enzyme activity**		
α-Glucosidase	76 ± 4 ^a^	129.5 ± 1.0 ^b^
α-Amylase	-	79.2 ± 1.6
Aldose-reductase	472 ± 9 ^a^	84 ± 6 ^b^

Values with different letters (^a,b^) in the same row are significantly different (*p* < 0.01). *^1^ IC_50_ value indicates the concentration of the extract that causes a 50% inhibition/scavenging in enzymatic activity/free radicals. *^2^ This value corresponds to the highest concentration tested: IC_50_ could not be determined and IC_25_ corresponded to 31 ± 2 µg/mL.

## Data Availability

Data are contained within the article.

## References

[1] Kumar M., Barbhai M.D., Hasan M., Punia S., Dhumal S., Radha, Rais N., Chandran D., Pandiselvam R., Kothakota A. (2022). Onion (*Allium Cepa* L.) Peels: A Review on Bioactive Compounds and Biomedical Activities. Biomed. Pharmacother..

[2] Črnivec I.G., Skrt M., Šeremet D., Sterniša M., Farčnik D., Štrumbelj E., Poljanšek A., Cebin N., Pogačnik L., Smole Možina S. (2021). Waste Streams in Onion Production: Bioactive Compounds, Quercetin and Use of Antimicrobial and Antioxidative Properties. Waste Manag..

[3] Marefati N., Ghorani V., Shakeri F., Boskabady M., Kianian F., Rezaee R., Boskabady M.H. (2021). A Review of Anti-Inflammatory, Antioxidant, and Immunomodulatory Effects of Allium Cepa and Its Main Constituents. Pharm. Biol..

[4] Ren F., Zhou S. (2021). Phenolic Components and Health Beneficial Properties of Onions. Agriculture.

[5] Sagar N.A., Pareek S. (2020). Antimicrobial Assessment of Polyphenolic Extracts from Onion (*Allium Cepa* L.) Skin of Fifteen Cultivars by Sonication-Assisted Extraction Method. Heliyon.

[6] Neeland I.J., Patel K.V. (2019). Diabetes: Key Markers of Injury and Prognosis. Biomarkers in Cardiovascular Disease.

[7] Santosh H.N., David C.M. (2017). Role of Ascorbic Acid in Diabetes Mellitus: A Comprehensive Review. J. Med. Radiol. Pathol. Surg..

[8] IDF (2021). IDF Diabetes Atlas.

[9] Mishra V., Nayak P., Sharma M., Albutti A., Alwashmi A.S.S., Aljasir M.A., Alsowayeh N., Tambuwala M.M. (2021). Emerging Treatment Strategies for Diabetes Mellitus and Associated Complications: An Update. Pharmaceutics.

[10] Kim K.T., Rioux L.E., Turgeon S.L. (2014). Alpha-Amylase and Alpha-Glucosidase Inhibition Is Differentially Modulated by Fucoidan Obtained from Fucus Vesiculosus and Ascophyllum Nodosum. Phytochemistry.

[11] Ahmed M.U., Ibrahim A., Dahiru N.J., Mohammed H.S. (2020). Alpha Amylase Inhibitory Potential and Mode of Inhibition of Oils from Allium Sativum (Garlic) and Allium Cepa (Onion). Clin. Med. Insights Endocrinol. Diabetes.

[12] Kim M.H., Jo S.H., Jang H.D., Lee M.S., Kwon Y.I. (2010). Antioxidant Activity and α-Glucosidase Inhibitory Potential of Onion (*Allium Cepa* L.) Extracts. Food Sci. Biotechnol..

[13] Benito-Román, Blanco B., Sanz M.T., Beltrán S. (2021). Freeze-Dried Extract from Onion (*Allium Cepa* Cv. Horcal) Skin Wastes: Extraction Intensification and Flavonoids Identification. Food Bioprod. Process..

[14] Trigueros E., Sanz M.T., Alonso-Riaño P., Beltrán S., Ramos C., Melgosa R. (2021). Recovery of the Protein Fraction with High Antioxidant Activity from Red Seaweed Industrial Solid Residue after Agar Extraction by Subcritical Water Treatment. J. Appl. Phycol..

[15] Atanasova A., Petrova A., Teneva D., Ognyanov M., Georgiev Y., Nenov N., Denev P. (2023). Subcritical Water Extraction of Rosmarinic Acid from Lemon Balm (*Melissa Officinalis* L.) and Its Effect on Plant Cell Wall Constituents. Antioxidants.

[16] Correia A., Silva A.M., Moreira M.M., Salazar M., Švarc-Gajić J., Brezo-Borjan T., Cádiz-Gurrea M.d.l.L., Carretero A.S., Loschi F., Dall’Acqua S. (2022). Salicornia Ramosissima: A New Green Cosmetic Ingredient with Promising Skin Effects. Antioxidants.

[17] Mandura Jarić A., Čikoš A., Pocrnić M., Aladić K., Jokić S., Šeremet D., Vojvodić Cebin A., Komes D. (2023). Teucrium Montanum L.—Unrecognized Source of Phenylethanoid Glycosides: Green Extraction Approach and Elucidation of Phenolic Compounds via NMR and UHPLC-HR MS/MS. Antioxidants.

[18] Boateng I.D. (2023). Mechanisms, Capabilities, Limitations, and Economic Stability Outlook for Extracting Phenolics from Agro-Byproducts Using Emerging Thermal Extraction Technologies and Their Combinative Effects. Food Bioprocess Technol..

[19] Cravotto C., Grillo G., Binello A., Gallina L., Olivares-vicente M., Herranz-lópez M., Micol V., Barrajón-catalán E., Cravotto G. (2022). Bioactive Antioxidant Compounds from Chestnut Peels through Semi-Industrial Subcritical Water Extraction. Antioxidants.

[20] Dunford N.T., Gumus Z.P., Gur C.S. (2022). Chemical Composition and Antioxidant Properties of Pecan Shell Water Extracts. Antioxidants.

[21] Benito-Román Ó., Blanco B., Sanz M.T., Beltrán S. (2020). Subcritical Water Extraction of Phenolic Compounds from Onion Skin Wastes (*Allium Cepa* Cv. Horcal): Effect of Temperature and Solvent Properties. Antioxidants.

[22] Trigueros E., Ramos C., Alonso-Riaño P., Beltrán S., Sanz M.T. (2023). Subcritical Water Treatment for Valorization of the Red Algae Residue after Agar Extraction: Scale-Up from Laboratory to Pilot Plant. Ind. Eng. Chem. Res..

[23] Chang C.-C., Yang M.-H., Wen H.-M., Chern J.-C. (2002). Estimation of Total Flavonoid Content in Propolis by Two Complementary Colometric Methods. J. Food Drug Anal..

[24] Nielsen S.S., Nielsen S.S. (2010). Phenol-Sulfuric Acid Method for Total Carbohydrates. Food Analysis Laboratory Manual.

[25] Bradford M.M. (1976). A Rapid and Sensitive Method for the Quantitation of Microgram Quantities of Protein Utilizing the Principle of Protein-Dry Bingin. Anal. Biochem..

[26] Ferreres F., Andrade C., Gomes N.G.M., Andrade P.B., Gil-Izquierdo A., Pereira D.M., Suksungworn R., Duangsrisai S., Videira R.A., Valentão P. (2021). Valorisation of Kitul, an Overlooked Food Plant: Phenolic Profiling of Fruits and Inflorescences and Assessment of Their Effects on Diabetes-Related Targets. Food Chem..

[27] Lee K.A., Kim K.T., Kim H.J., Chung M.S., Chang P.S., Park H., Pai H.D. (2014). Antioxidant Activities of Onion (*Allium Cepa* L.) Peel Extracts Produced by Ethanol, Hot Water, and Subcritical Water Extraction. Food Sci. Biotechnol..

[28] Kim, Ko M.J., Park C.H., Chung M.S. (2022). Application of Pulsed Electric Field as a Pre-Treatment for Subcritical Water Extraction of Quercetin from Onion Skin. Foods.

[29] Dhanya R. (2022). Quercetin for Managing Type 2 Diabetes and Its Complications, an Insight into Multitarget Therapy. Biomed. Pharmacother..

[30] Rocha L., Neves D., Valentão P., Andrade P.B., Videira R.A. (2020). Adding Value to Polyvinylpolypyrrolidone Winery Residue: A Resource of Polyphenols with Neuroprotective Effects and Ability to Modulate Type 2 Diabetes-Relevant Enzymes. Food Chem..

[31] Li X., Wang X., Chen D., Chen S. (2011). Antioxidant Activity and Mechanism of Protocatechuic Acid in Vitro. Funct. Foods Health Dis..

[32] Kumari N., Kumar M., Radha, Lorenzo J.M., Sharma D., Puri S., Pundir A., Dhumal S., Bhuyan D.J., Jayanthy G. (2022). Onion and Garlic Polysaccharides: A Review on Extraction, Characterization, Bioactivity, and Modifications. Int. J. Biol. Macromol..

[33] Khalili S., Saeidi Asl M.R., Khavarpour M., Vahdat S.M., Mohammadi M. (2022). Comparative Study on the Effect of Extraction Solvent on Total Phenol, Flavonoid Content, Antioxidant and Antimicrobial Properties of Red Onion (*Allium Cepa*). J. Food Meas. Charact..

[34] Papoutsis K., Zhang J., Bowyer M.C., Brunton N., Gibney E.R., Lyng J. (2021). Fruit, Vegetables, and Mushrooms for the Preparation of Extracts with α-Amylase and α-Glucosidase Inhibition Properties: A Review. Food Chem..

[35] Aleixandre A., Gil J.V., Sineiro J., Rosell C.M. (2022). Understanding Phenolic Acids Inhibition of α-Amylase and α-Glucosidase and Influence of Reaction Conditions. Food Chem..

[36] Ruivo Da Silva M.G., Skrt M., Komes D., Poklar Ulrih N., Pogačnik L. (2020). Enhanced Yield of Bioactivities from Onion (*Allium Cepa* L.) Skin and Their Antioxidant and Anti-α-Amylase Activities. Int. J. Mol. Sci..

[37] Sun L., Warren F.J., Gidley M.J. (2019). Natural Products for Glycaemic Control: Polyphenols as Inhibitors of Alpha-Amylase. Trends Food Sci. Technol..

[38] Liu X., Wang Y., Zhang J., Yan L., Liu S., Taha A.A., Wang J., Ma C. (2020). Subcritical Water Extraction of Phenolic Antioxidants with Improved α-Amylase and α-Glucosidase Inhibitory Activities from Exocarps of Castanea Mollissima Blume. J. Supercrit. Fluids.

[39] Ti Y., Wang W., Wang X., Ban Y., Wang P., Zhang Y., Song Z. (2022). Pumpkin Polysaccharide Extracted by Subcritical Water: Physicochemical Characterization and Anti-Diabetic Effects in T2DM Rats. Mol. Nutr. Food Res..

[40] Wang S.T., Dan Y.Q., Zhang C.X., Lv T.T., Qin Z., Liu H.M., Ma Y.X., He J.R., Wang X. (2022). De Structures and Biological Activities of Proanthocyanidins Obtained from Chinese Quince by Optimized Subcritical Water-Ethanol Extraction. J. Food Meas. Charact..

[41] Wu T., Luo J., Xu B. (2015). In Vitro Antidiabetic Effects of Selected Fruits and Vegetables against Glycosidase and Aldose Reductase. Food Sci. Nutr..

[42] Yang J., Meyers K.J., Van Der Heide J., Rui H.L. (2004). Varietal Differences in Phenolic Content and Antioxidant and Antiproliferative Activities of Onions. J. Agric. Food Chem..

[43] Jocković N., Fischer W., Brandsch M., Brandt W., Dräger B. (2013). Inhibition of Human Intestinal α-Glucosidases by Calystegines. J. Agric. Food Chem..

